# Research and Application of Deep Learning Models with Multi-Scale Feature Fusion for Lesion Segmentation in Oral Mucosal Diseases

**DOI:** 10.3390/bioengineering11111107

**Published:** 2024-11-02

**Authors:** Rui Zhang, Miao Lu, Jiayuan Zhang, Xiaoyan Chen, Fudong Zhu, Xiang Tian, Yaowu Chen, Yuqi Cao

**Affiliations:** 1 Zhejiang Provincial Key Laboratory of Internet Multimedia Technology, College of Biomedical Engineering & Instrument Science, Zhejiang University, Hangzhou 310027, China; zhangrui46@zju.edu.cn (R.Z.); tianx@zju.edu.cn (X.T.); yaowuchen@zju.edu.cn (Y.C.); 2 Key Laboratory of Oral Biomedical Research of Zhejiang Province, Cancer Center of Zhejiang University, Stomatology Hospital, School of Stomatology, Zhejiang University School of Medicine, Zhejiang Provincial Clinical Research Center for Oral Diseases, Engineering Research Center of Oral Biomaterials and Devices of Zhejiang Province, Hangzhou 310053, China; ortho_chenxy@zju.edu.cn (X.C.); zfd@zju.edu.cn (F.Z.); 3 Life Health Innovation and Entrepreneurship Center, Institute of Wenzhou, Zhejiang University, Wenzhou 325000, China; 4 State Key Laboratory of Industrial Control Technology, College of Control Science and Engineering, Zhejiang University, Hangzhou 310027, China; 22432005@zju.edu.cn (M.L.); 12432086@zju.edu.cn (J.Z.)

**Keywords:** deep learning, oral mucosal disease, semantic segmentation, oral mucosal disease, SegFormer model, lesion detection, computer-aided diagnosis

## Abstract

Given the complexity of oral mucosal disease diagnosis and the limitations in the precision of traditional object detection methods, this study aims to develop a high-accuracy artificial intelligence-assisted diagnostic approach based on the SegFormer semantic segmentation model. This method is designed to automatically segment lesion areas in white-light images of oral mucosal diseases, providing objective and quantifiable evidence for clinical diagnosis. This study utilized a dataset of oral mucosal diseases provided by the Affiliated Stomatological Hospital of Zhejiang University School of Medicine, comprising 838 high-resolution images of three diseases: oral lichen planus, oral leukoplakia, and oral submucous fibrosis. These images were annotated at the pixel level by oral specialists using Labelme software (v5.5.0) to construct a semantic segmentation dataset. This study designed a SegFormer model based on the Transformer architecture, employed cross-validation to divide training and testing sets, and compared SegFormer models of different capacities with classical segmentation models such as UNet and DeepLabV3. Quantitative metrics including the Dice coefficient and mIoU were evaluated, and a qualitative visual analysis of the segmentation results was performed to comprehensively assess model performance. The SegFormer-B2 model achieved optimal performance on the test set, with a Dice coefficient of 0.710 and mIoU of 0.786, significantly outperforming other comparative algorithms. The visual results demonstrate that this model could accurately segment the lesion areas of three common oral mucosal diseases. The SegFormer model proposed in this study effectively achieves the precise automatic segmentation of three common oral mucosal diseases, providing a reliable auxiliary tool for clinical diagnosis. It shows promising prospects in improving the efficiency and accuracy of oral mucosal disease diagnosis and has potential clinical application value.

## 1. Introduction

Oral health issues have gained significant attention in society in recent years. According to the World Health Organization’s Global Oral Health Status Report 2022 [1], nearly 3.5 billion people worldwide suffer from oral diseases, with 75% of them coming from middle-income countries. The early diagnosis of oral diseases can improve cure rates, reduce treatment difficulty and costs, and prevent the occurrence of complications. Among oral diseases, mucosal diseases are both common and complex, mainly including oral lichen planus, oral leukoplakia, and oral submucous fibrosis. In recent years, due to the prevalence of unhealthy lifestyles such as long periods of staying up late, excessive smoking and drinking, and imbalanced diets, the number of young people with oral cancer has significantly increased. In 2020, approximately 840,000 new cases of oral cancer were reported globally, resulting in around 420,000 deaths [2,3,4]. These statistics underscore the importance of early diagnosis in improving patient outcomes for oral mucosal diseases.

In the identification and diagnosis of oral mucosal diseases, traditional manual recognition has many limitations. First, the diagnostic results are highly dependent on the professional knowledge and clinical experience of doctors, which are easily influenced by subjective factors, making it difficult to ensure the accuracy and consistency of diagnosis. Secondly, the training cycle for high-level doctors is long and costly, and it is difficult to cultivate in areas with a shortage of medical resources. With the rapid development of artificial intelligence technology, disease recognition methods based on semantic segmentation have shown significant advantages. Semantic segmentation models use deep learning techniques to train and learn from a large number of medical image data and can automatically extract and learn characteristic information of diseases, realizing the accurate identification and localization of diseases. This method overcomes the subjectivity and uncertainty of manual recognition and improves the objectivity and accuracy of diagnosis. Moreover, semantic segmentation models can continuously learn and be optimized to continuously improve the performance of disease identification, reducing misdiagnosis and missed diagnosis. Deep learning techniques for image segmentation predominantly encompass models like U-Net, SegNet, and DeepLab [5,6,7]. U-Net features a symmetric encoder–decoder structure, optimizing performance with fewer data instances by using extensive data augmentation. SegNet utilizes an encoder–decoder architecture with upsampled feature maps for precise boundary delineation. DeepLab employs atrous convolutions and fully connected CRFs to capture multi-scale information and refine segmentation edges. Each method is designed for specific scenarios like medical imaging (U-Net), scene understanding (SegNet), and large-scale contextual information (DeepLab), showcasing varied strategies to address segmentation challenges.

Vision Transformers (ViTs) have expanded into several state-of-the-art models including Swin Transformer, which uses shifted windows for efficient self-attention [8,9]. TransUNet integrates Transformer layers within a U-Net structure, while Swin-Unet further adapts the Swin Transformer for medical image segmentation, enhancing feature extraction capabilities [10,11]. These models leverage the Transformer’s ability to handle long-range dependencies, making them highly effective across various vision tasks beyond simple classification.

Currently, research on the application of deep learning methods for the analysis of oral mucosal disease images mainly focuses on disease classification and lesion area target detection [12,13,14,15,16,17,18]. Lin et al. used the high-resolution deep learning method HRNet to detect oral cancer and achieved a sensitivity of 83.0% and specificity of 96.6% [13]. Warin et al. utilized DenseNet121 and Faster R-CNN to obtain the binary image classification and object detection results of OSCC and normal oral mucosa, and the AUC and ROC curve were 0.99 and 0.79, respectively [15]. Moreover, automatic oral cancer detection has also been extensively studied [19,20,21,22,23,24,25]. Yang et al. developed a CNN-based model for OSCC diagnosis and made further comparisons of model and human performance [20]. Deif et al. employed four common deep neural networks, VGG16, AlexNet, ResNet50, and InceptionV3, for the feature extraction of OSCC and combined them with machine learning methods to achieve an accuracy of 96.3% [22]. A few studies have classified and detected lesions for several types of oral mucosal diseases [26] and oral cancer [27,28]. However, these studies are mostly limited to singular disease target detection, with relatively narrow detection categories, and the detection output results are usually a set of rectangular box coordinates and corresponding category labels, with very limited detection accuracy. At present, there is still a gap in the research on high-precision lesion segmentation for multiple oral mucosal diseases.

This research introduces an approach to enhance lesion segmentation in oral mucosal disease images through advanced deep learning techniques. By employing sophisticated algorithms, this study uniquely automates the extraction of lesion-specific features, facilitating precise, pixel-level segmentation across diverse types of oral mucosal diseases. Anticipated to markedly boost diagnostic accuracy and streamline treatment processes, this method stands out for its potential to assist clinical practices, showing good clinical application value and prospects.

The organization of this paper is as follows: Section 2 introduces the materials and methods used in this study. Section 2.1 provides a detailed description of the dataset collection and annotation process. Section 2.2 describes the structure of the SegFormer semantic segmentation model. Section 2.3 discusses the model’s training configuration parameters. Section 2.4 covers data preprocessing and augmentation methods during training. Section 2.5 introduces the evaluation metrics used in the semantic segmentation experiments. Section 3 presents the results of the semantic segmentation experiments. Section 4 discusses the experiments and research findings. Section 5 summarizes this entire work.

## 2. Materials and Methods

### 2.1. Research Materials

#### 2.1.1. Data Source and Selection

**Figure 1:**
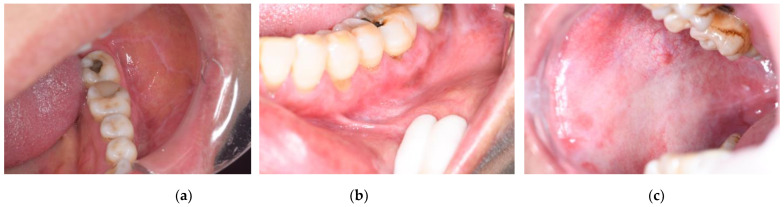
Samples of the dataset. (**a**) OLP; (**b**) OLK; (**c**) OSF.

Figure 1 shows some samples in the dataset. The dataset used in this study was collected from oral mucosal disease patients between 2020 and 2022. Patients were aged from 18 to 70 years old, encompassing both males and females. The cases provide comprehensive pre- and post-treatment intraoral white-light photographs, supplemented by pathology reports. The oral diagnoses included oral leukoplakia (OLK), oral lichen planus (OLP), and oral submucous fibrosis (OSF). Images of the cases with inadequate image quality, including blurred, unclear, or improperly exposed, were excluded.

A total of 838 images were collected, including 523 images of OLP, 201 images of OLK, and 114 images of OSF. The image resolution covers three specifications: 8256 × 5504, 6192 × 4128, and 6000 × 4000. The dataset was randomly divided into training, validation, and test sets in a 6:2:2 ratio.

#### 2.1.2. Lesion Site Annotation

All training data were annotated by experienced oral physicians, including those of OLP, OLK, and OSF. Using the LabelMe software (v5.5.0), the disease contours were accurately marked with smooth and continuous curves, and the corresponding diagnosis results were recorded.

#### 2.1.3. Consistency Check and Annotation

Data Annotation and Consistency Validation: Two experienced clinicians independently annotated the acquired training data, delineating diagnoses and disease boundaries for each case, followed by a comparative analysis and consistency assessment. In instances of diagnostic uncertainty, senior consultants were consulted to establish definitive clinical diagnoses, thereby ensuring inter-observer reliability. To assess intra-observer reliability, the same clinicians re-annotated all training data after a two-week interval. Datasets that successfully passed the consistency validation were deemed suitable for inclusion in the machine learning model development. Cases failing to meet consistency criteria underwent additional annotation by a third experienced clinician. Persistently inconsistent datasets were excluded from this study to maintain data integrity.

### 2.2. Construction of SegFormer Semantic Segmentation Model Based on Transformer

#### 2.2.1. Encoder Design

**Figure 2:**
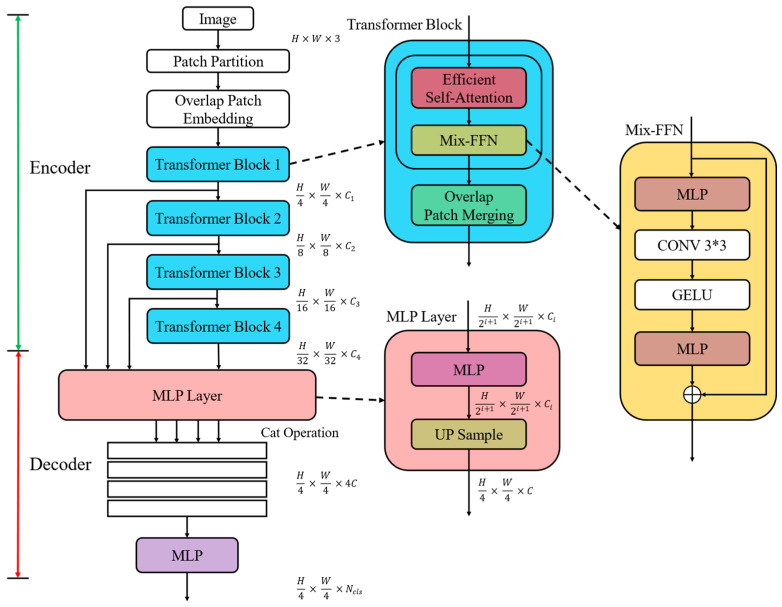
The overall structure of the SegFormer semantic segmentation model.

This study employs the SegFormer model [29], which is based on the Transformer architecture, to perform lesion semantic segmentation on oral mucosal disease datasets. The core framework of the SegFormer model comprises two key components: an encoder and a decoder. The encoder is responsible for extracting and encoding input image features, generating high-level feature representations, while the decoder processes these extracted features to produce the final segmentation results. The overall structure of SegFormer is illustrated in Figure 2.

The SegFormer encoder design incorporates the image patch partitioning concept from Vision Transformer [8], adopting a sequential input structure. The encoding process involves the following steps:

1. Image patch partitioning—Dividing the input image into multiple equal-sized patches, typically 4 × 4 pixels each;
2. Sequence processing—Converting image patches into a sequence of vector representations through learnable linear mappings;
3. Problem transformation—Recasting computer vision tasks as sequence input problems, leveraging Transformer’s global information modeling capabilities;
4. Position encoding—Introducing position information for image patches to enhance spatial awareness and improve segmentation accuracy.

This design effectively combines the Transformer’s strength in sequence processing with its ability to handle 2D image data, making it particularly suitable for complex semantic segmentation tasks such as oral mucosal lesion detection.

While maintaining the serial stacking of Transformer modules, SegFormer incorporates the multi-scale information extraction strategy from Swin Transformer [9]. This approach progressively reduces spatial resolution while increasing feature channels after each Transformer module, resulting in more abstract and rich high-level features. Assuming an input image size of H × W × 3, the encoder’s serial feature extraction process produces a series of feature maps with dimensions (H/2i + 1 × W/2i + 1 × Ci), where i = 1, 2, 3, 4, and Ci denotes the increasing channel number.

SegFormer enhances the Swin Transformer’s patch merging operation by incorporating an overlapping patch merging technique. This approach allows for pixel overlap between adjacent patches, effectively maintaining pixel continuity and achieving a better balance between global information fusion and local detail preservation.

SegFormer also refined the standard Transformer self-attention mechanism by introducing an efficient computation method. Given an input sequence of size N × C, where N is the sequence length, and C is the vector dimension, the efficient self-attention mechanism uses a decay factor R to compress the input sequence length.

K′ = Reshape (N/R, C∙R) (K),(1)

K = Linear (C∙R, C) (K′)(2)

The two-step process first adjusts the input sequence K from N × C to N/R × (C∙R), then projects it back to N/R × C through a learnable linear layer. This reduces the computational complexity of self-attention from O(N^2^) to O(N^2^/R), significantly boosting efficiency.

**Figure 3:**
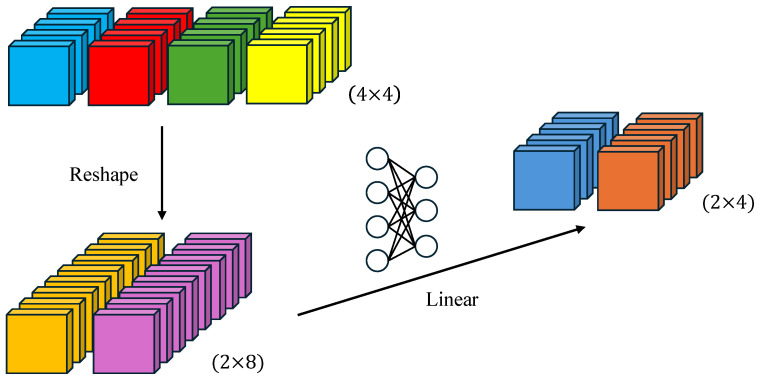
A schematic of the efficient self-attention mechanism.

Figure 3 illustrates this efficient self-attention mechanism for an input size of 4 × 4 with a decay factor R = 2. The improved self-attention mechanism not only preserves the Transformer model’s capability to capture long-range dependencies, but it also substantially reduces computational complexity, enabling SegFormer to more efficiently process high-resolution images. This refinement is particularly well suited for precise lesion segmentation in the context of oral mucosal diseases.

#### 2.2.2. Decoder Design

The SegFormer decoder is designed to effectively fuse multi-scale features extracted by the encoder, achieving high-precision semantic segmentation. It utilizes a multilayer perceptron (MLP) structure, encompassing feature projection, upsampling, feature concatenation, and final output generation. The decoding process can be formalized as follows, where F_i_ represents the input feature map from the encoder.

F_i_′ = Linear (C_i_, C) (F_i_), (3)

F_i_″ = Upsample (H/4, W/4) (F_i_′), (4)

F = Linear (4C, C) (Concat (F_i_″)), (5)

M = Linear (C, N_cls_) (F) (6)

This process includes the following:

1. Feature projection (Equation (3))—The multi-scale feature representations, initially possessing diverse channel dimensions, are linearly transformed to a channel number C. This process facilitates the standardization of feature dimensionality across the different scales.
2. Feature upsampling (Equation (4))—Feature maps with identical channel numbers but differing resolutions are upsampled to a uniform size of one-quarter of the original image dimensions (H/4 × W/4), ensuring spatial resolution consistency.
3. Feature fusion (Equation (5))—Feature maps of equivalent resolution and channel count are initially concatenated along the channel dimension. Subsequently, a linear layer projects the channel count back to C, facilitating the effective integration of multi-scale features.
4. Output generation (Equation (6))—A linear layer projects the channel dimension of the fused feature map to the number of categories N_cls_, yielding the final segmentation mask with dimensions (H/4 × W/4 × N_cls_).

A decoder based on multilayer perceptrons (MLPs) can simplify model design and reduce computational complexity compared to convolutional neural networks. This MLP-based architecture is able to effectively integrate multi-scale feature information, leading to improved segmentation accuracy. Additionally, the MLP structure is more computationally efficient than complex convolutions, making it easy to adjust and optimize for specific tasks.

The MLP-based decoder design is particularly well suited for the fine-grained segmentation of oral mucosal lesions. By leveraging the multi-scale features extracted by the encoder, this approach can generate high-quality segmentation results. In this way, the SegFormer model is able to achieve efficient semantic segmentation while maintaining strong performance.

Overall, the simplicity and flexibility of the MLP-based decoder, combined with its ability to exploit multi-scale representations, make it a promising architecture for medical image analysis tasks like lesion segmentation.

#### 2.2.3. GELU Activation Function

SegFormer utilizes the Gaussian Error Linear Unit (GELU) [30] as its activation function. The GELU adjusts activation probabilities based on input magnitudes, introducing a regularization effect and enhancing the model’s generalization capability. The GELU function is defined as follows:

GELU(x) = xP(X ≤ x) = xΦ(x)(7)

where Φ(x) is the cumulative distribution function of the standard normal distribution. In practice, an approximation is used for computational efficiency.

GELU(x) = 0.5x(1 + tanh[(2/π)^1/2^ (x + 0.44715x^3^)]) (8)

The GELU function enhances the model’s expressive ability, helps alleviate the gradient vanishing problem, and introduces a slight regularization effect. Its adaptability to inputs of different scales is particularly beneficial for processing multi-scale features in the SegFormer model.

By incorporating the GELU activation function, SegFormer improves feature extraction effectiveness and overall model performance while maintaining model complexity. This is crucial for accurately segmenting lesions in oral mucosal diseases, enabling efficient semantic segmentation with high precision.

### 2.3. SegFormer Training Configuration

This study employs three variants of SegFormer, B0, B1, and B2, which exhibit an increasing trend in model capacity. The specific configurations of these variants are presented in Table 1.

The encoder vector dimension serves as an indicator of the model’s feature extraction capability following each downsampling operation. While the post-downsampling resolution remains consistent across different models, the variation in channel numbers reflects their differing capacities to capture feature complexity. The module depth denotes the number of stacked Transformer modules within the encoder. It is noteworthy that increasing the stacking depth does not alter the feature map dimensions; rather, it reduces resolution and augments vector dimensionality through patch fusion at each module’s output. The number of attention heads influences the diversity of feature extraction, whereas the decoder vector dimension determines the information richness in feature fusion.

To enhance model performance, this study implements a comprehensive set of training strategies. Initially, the model is initialized using weights pre-trained on the ImageNet1k dataset [31], a technique known to accelerate convergence and improve generalization capabilities. The optimization process utilizes the AdamW algorithm, with an initial learning rate of 1 × 10^−4^ and a weight decay coefficient of 1 × 10^−2^. This configuration is conducive to improving model convergence and mitigating overfitting. The learning rate is modulated using a cosine annealing schedule, which dynamically adjusts the rate to maintain an optimal balance between exploration and exploitation throughout the training process. The loss function incorporates a combination of Focal loss and Dice loss. The former addresses class imbalance issues, while the latter optimizes segmentation boundary accuracy, resulting in a comprehensive enhancement in segmentation performance.

These meticulously designed model variants and training strategies are intended to fully leverage SegFormer’s potential in oral mucosal disease segmentation tasks. Through the adjustment of model capacities and the application of efficient training techniques, this study aims to identify the most suitable configuration for this specific task, with the ultimate goal of achieving an optimal balance between accuracy and computational efficiency.

### 2.4. Data Preprocessing and Augmentation

To enhance the generalization capability and robustness of the SegFormer model in segmenting oral mucosal diseases, a series of meticulously designed data preprocessing and augmentation strategies were implemented. These strategies were devised to simulate real-world image variations, thereby improving the model’s adaptability to diverse image conditions.

The preprocessing phase began with image standardization, normalizing pixel values to the [0, 1] range to mitigate brightness and contrast disparities across images. Subsequently, the following data augmentation techniques were employed:

1. Random cropping—First, 512 × 512-pixel regions were randomly extracted from original images, encouraging the model to focus on local features and increasing training sample diversity.
2. Random flipping—Images were horizontally or vertically flipped with 50% probability, promoting the model’s ability to recognize features in various orientations.
3. Random rotation—Images were rotated within a [−10°, 10°] range, simulating minor variations in capture angles and enhancing the model’s resilience to slight perspective changes.
4. Brightness and contrast adjustment—Image brightness and contrast were randomly modified within the range of [0.8, 1.2], facilitating the model’s adaptation to varying lighting conditions. Furthermore, random adjustments in hue [−0.05, 0.05], saturation [0.9, 1.1], and color balance [−0.05, 0.05] were introduced to emulate color variations arising from different imaging devices and environments.

These augmentation techniques preserved the original semantic information while substantially expanding the effective training sample size. By introducing these controlled random variations, the model was compelled to learn more robust and generalized feature representations, potentially improving its performance on unseen test data.

This comprehensive data preprocessing and augmentation protocol provided the SegFormer model with a rich, diverse training dataset. Consequently, it mitigated overfitting issues and enhanced the model’s generalization capability and reliability in real-world clinical applications.

### 2.5. Semantic Segmentation Evaluation Metrics

The present research utilizes several widely adopted evaluation metrics in the domain of semantic segmentation, including the Dice coefficient, mean Intersection over Union (mIoU), mean pixel accuracy (mPA), and precision. The Dice coefficient, in particular, stands as the most frequently employed evaluation metric in semantic segmentation, serving to quantify the similarity between predicted segmentation results and ground truth labels. This metric primarily assesses the classification accuracy of foreground pixels, with its practical implementation involving the calculation of the mean Dice value across all categories. The Dice coefficient is mathematically expressed as follows:

Dice = 2 × |Prediction ∩ Truth|/(|Prediction| + |Truth|) = 2 × TP/(2 × TP + FP + FN)(9)

In this equation, TP, FP, and FN denote the numbers of true positive, false positive, and false negative pixels, respectively.

Another crucial evaluation metric is the mean Intersection over Union (mIoU), which is computed as the average of the IoU (Intersection over Union) values across all categories. The IoU is calculated using the following formula:

IoU = |Prediction ∩ Truth|/|Prediction ∪ Truth| = TP/(TP + FP + FN)(10)

The mean pixel accuracy (mPA) represents the average of the pixel accuracy (PA) values across all categories. PA serves as an intuitive evaluation metric in semantic segmentation tasks and is defined as follows:

PA = (TP + TN)/(TP + TN + FP + FN)(11)

These evaluation metrics collectively provide a comprehensive assessment of the segmentation model’s performance, offering insights into its accuracy and effectiveness from various perspectives.

## 3. Results

This study conducted a comparative analysis of three SegFormer models with var-ying capacities against classic semantic segmentation models (U-Net, DeepLabV3+, PSPNet) and the general high-resolution model HRNet in the context of oral disease segmentation. Additionally, we compared various Transformer-based semantic segmentation models, including the UPerNet network using Swin Transformer as the feature extraction backbone, as well as Segmenter, MaskFormer, and Swin-Unet. We quantified the parameter count and computational complexity (FLOPs) for each model, with the results presented in Table 2.

The experimental results demonstrate the superior performance of SegFormer models in this task. While the lightweight SegFormer-B0 showed competitive results, SegFormer-B1 and SegFormer-B2 significantly outperformed other models across key performance metrics (Dice, mIoU, and precision). In particular, the SegFormer-B2 model showed marked improvements over established segmentation networks like U-Net and DeepLabV3+, as well as other semantic segmentation models based on Vision Transformer.

The optimal SegFormer-B2 model achieved impressive results with a Dice coefficient of 0.710, mIoU of 0.786, mean pixel accuracy of 0.879, and precision of 0.886. Remarkably, the B2 model achieved these results with a parameter count comparable to or lower than the baseline U-Net while maintaining a computationally efficient profile. Other semantic segmentation models based on Vision Transformer, such as Segmenter and Swin-Unet, generally have a higher number of parameters and computational load. In contrast, SegFormer-B2 utilizes efficient self-attention, which reduces the parameter count while maintaining model performance, thereby offering a clear advantage.

**Figure 4:**
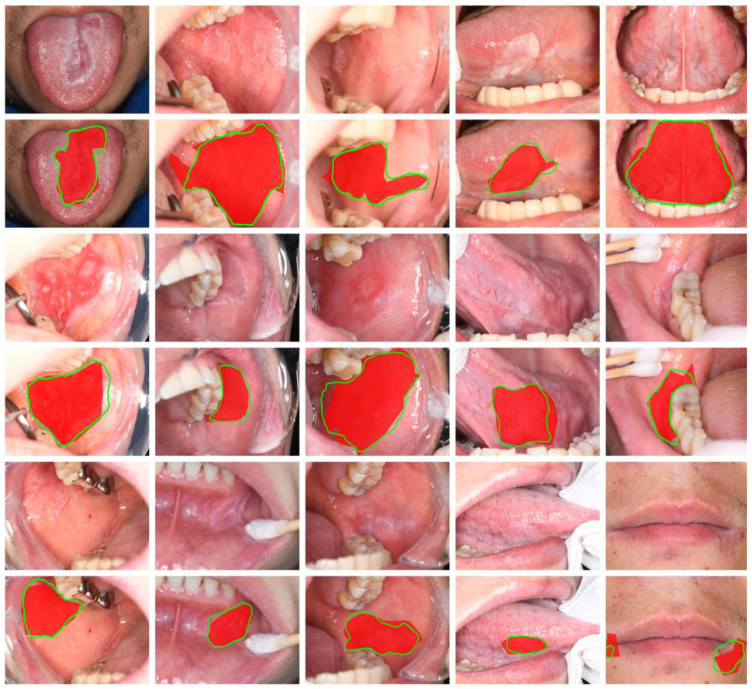
SegFormer semantic segmentation visualization of oral mucosal lesions.

To visualize the model’s performance, we applied the trained SegFormer-B2 model to segment oral disease images from the test set. Figure 4 illustrates this performance, presenting five images per row, each containing examples of three categories (three OLP, one OLK, one OSF). The figure displays the original image, ground truth lesion area (filled in red), and the predicted segmentation mask (outlined in green). This visual analysis confirms the SegFormer-B2 model’s ability to accurately delineate lesion areas across three common oral diseases (OLP, OLK, OSF), providing precise localization and morphological information.

It can be observed that compared to the ground truth annotations in the red areas, SegFormer-B2 provides more precise predictions of lesion edges, offering information about the shape, size, and category of the lesions, which makes it highly referential.

## 4. Discussion

This study introduces an innovative deep learning-based method for segmenting lesions in oral mucosal disease images. The proposed approach demonstrated excellent performance, achieving a Dice coefficient of 0.710 and a mean Intersection over Union (mIoU) of 0.786 in experimental evaluations. In contrast to previous research, such as the work by Gizem et al., which treated all annotated data as a single class during model training [32], our method effectively differentiates between various types of oral mucosal diseases. Moreover, it shows marked improvements in segmentation accuracy compared to the multi-task learning approach, for instance, segmentation proposed by Guan et al.

This study examines the use of the SegFormer deep learning algorithm for semantic segmentation in delineating lesions of oral malignant diseases, providing detailed contours of the lesion areas, which aids doctors in identifying the type, size, and severity of the lesions. This contrasts with other studies in which image classification only provides a general disease category for the entire image without the location information of the lesions—thus offering limited assistance to the diagnostic process. Some studies use object detection algorithms to identify the approximate location of lesions but lack detailed contour information.

The superior performance of the SegFormer model can be attributed to several key factors:

1. Multi-scale feature fusion—The model’s encoder–decoder architecture enables the simultaneous capture of local details and global semantic information, enhancing segmentation accuracy and comprehensiveness;
2. Overlapping patch merging—This novel technique maintains local pixel continuity, significantly improving segmentation precision in edge regions and yielding more refined results;
3. Gaussian Error Linear Unit (GELU) activation function—The incorporation of the GELU provides a regularization effect and enhances the model’s generalization capabilities, allowing for better adaptation to diverse data distributions and task scenarios.

Our experiments comparing U-Net, PSPNet, DeepLabV3+, HRNet, and Vision Transformer-based models and SegFormer models of varying capacities showed that increasing the input resolution and model capacity can promote segmentation performance to some extent. To enhance the coefficient and precision of the model in future applications, it is crucial to collect a larger quantity of high-quality annotated datasets and fine-tune the models. Moreover, further optimizing the model architecture can be achieved by increasing the input image resolution and improving multi-scale feature fusion techniques. Higher input resolution preserves more low-level semantic information, while more sophisticated methods of multi-scale feature fusion can learn more efficiently from features at various resolutions, allowing the model to exhibit superior performance at the same capacity.

Despite these positive outcomes, this study has certain limitations. The dataset, while sourced from a tertiary specialized oral hospital in Zhejiang Province, is relatively limited in scale and geographical diversity. This constraint may impact the model’s ability to comprehensively capture disease characteristics across different populations. Additionally, the lack of external datasets for testing the semantic segmentation model’s generalization limits our ability to fully assess its robustness and transferability. Although the deep learning process included corrections for variations in camera angles and lighting conditions, the absence of validation on external datasets precludes a comprehensive evaluation of the model’s adaptability. Additionally, the model was only trained on three types of oral malignant disease datasets, which may lead to poor or erroneous segmentation for other malignant or benign diseases. Furthermore, the interpretability of deep learning models is subpar, requiring further consideration and research for practical application in clinical diagnostic support.

In clinical applications, automated lesion segmentation in oral mucosal disease images presents a promising approach to enhance oral disease diagnosis and treatment. Compared to subjective manual assessments, this method provides greater standardization and consistency by utilizing objective image features. It can assist clinicians in rapidly and objectively evaluating lesion location, size, and morphological characteristics, potentially detecting subtle abnormalities that might be overlooked by visual inspection alone. This capability could significantly enhance early disease detection and intervention. Moreover, the systematic segmentation of lesion areas across large-scale datasets can contribute to a more comprehensive understanding of oral mucosal disease imaging features, supporting in-depth investigations into disease mechanisms. The quantitative analysis of lesion areas enables the precise monitoring of disease progression, providing valuable insights for treatment planning and adjustment.

## 5. Conclusions

In conclusion, the SegFormer-based oral mucosal disease image lesion segmentation method developed in this study demonstrates significant performance advantages and broad clinical application potential. As artificial intelligence technologies continue to advance and integrate more deeply with clinical practice, such intelligent diagnostic support tools are expected to play an increasingly crucial role in enhancing the quality and efficiency of oral healthcare, ultimately leading to more precise and effective patient care.

## Figures and Tables

**Table 1:** SegFormer model configurations.

Model	Input Resolution	Encoder Dimensions	Encoder Depth	Attention Heads	Decoder Dimension
B0	[512, 512]	[32, 64, 160, 256]	[2, 2, 2, 2]	[1, 2, 5, 8]	256
B1	[512, 512]	[64, 128, 320, 512]	[2, 2, 2, 2]	[1, 2, 5, 8]	256
B2	[512, 512]	[64, 128, 320, 512]	[3, 4, 6, 3]	[1, 2, 5, 8]	768

**Table 2:** Semantic segmentation task results.

Model	Backbone	Params	FLOPs	Dice	mIoU	mPA	Precision
U-Net	VGG	24.82 M	451.71 G	0.636	0.683	0.832	0.784
U-Net	ResNet50	43.93 M	184.13 G	0.665	0.728	0.867	0.813
PSPNet	MobileNetV2	2.38 M	6.03 G	0.627	0.692	0.852	0.765
PSPNet	ResNet50	46.71 M	118.42 G	0.670	0.725	0.862	0.819
DeepLabV3+	MobileNetV2	5.82 M	52.88 G	0.667	0.716	0.830	0.817
DeepLabV3+	Xception	166.85 M	54.71 G	0.680	0.682	0.892	0.749
HRNet	W18	9.64 M	32.81 G	0.669	0.737	0.853	0.824
HRNet	W32	29.54 M	79.93 G	0.698	0.745	0.844	0.841
UPerNet	Swin-B	120.20 M	82.10 G	0.687	0.756	0.853	0.806
Segmenter	ViT-L	333.08 M	665.44 G	0.704	0.752	0.865	0.758
MaskFormer	Swin-T	41.76 M	98.51 G	0.705	0.735	0.815	0.833
Swin-Unet	Swin-L	335.80 M	200.02 G	0.699	0.670	0.736	0.879
SegFormer	MiT-B0	3.72 M	13.55 G	0.659	0.758	0.864	0.844
SegFormer	MiT-B1	13.68 M	26.50 G	0.698	0.774	0.859	0.874
SegFormer	MiT-B2	27.35 M	113.45 G	0.710	0.786	0.876	0.886

## Data Availability

The data supporting this study’s findings are available from the corresponding author upon reasonable request.
